# Serum Amyloid A1 (SAA1) Revisited: Restricted Leukocyte-Activating Properties of Homogeneous SAA1

**DOI:** 10.3389/fimmu.2020.00843

**Published:** 2020-05-14

**Authors:** Sara Abouelasrar Salama, Mirre De Bondt, Mieke De Buck, Nele Berghmans, Paul Proost, Vivian Louise Soares Oliveira, Flavio A. Amaral, Mieke Gouwy, Jo Van Damme, Sofie Struyf

**Affiliations:** ^1^Laboratory of Molecular Immunology, Department of Microbiology, Immunology and Transplantation, Rega Institute for Medical Research, KU Leuven, Leuven, Belgium; ^2^Laboratório de Imunofarmacologia, Departamento de Bioquímica e Imunologia, Instituto de Ciências Biológicas, Universidade Federal de Minas Gerais, Belo Horizonte, Brazil

**Keywords:** SAA, neutrophils, FPR2, chemotaxis, chemokines, ROS, MMP-9, macrophages

## Abstract

Infection, sterile injury, and chronic inflammation trigger the acute phase response in order to re-establish homeostasis. This response includes production of positive acute phase proteins in the liver, such as members of the serum amyloid A (SAA) family. In humans the major acute phase SAAs comprise a group of closely related variants of SAA1 and SAA2. SAA1 was proven to be chemotactic for several leukocyte subtypes through activation of the G protein-coupled receptor FPRL1/FPR2. Several other biological activities of SAA1, such as cytokine induction, reported to be mediated via TLRs, have been debated recently. Especially commercial SAA1, recombinantly produced in *Escherichia coli*, was found to be contaminated with bacterial products confounding biological assays performed with this rSAA1. We purified rSAA1 by RP-HPLC to homogeneity, removing contaminants such as lipopolysaccharides, lipoproteins and formylated peptides, and re-assessed several biological activities attributed to SAA1 (chemotaxis, cytokine induction, MMP-9 release, ROS generation, and macrophage differentiation). The homogeneous rSAA1 (hrSAA1) lacked most cell-activating properties, but its leukocyte-recruiting capacity *in vivo* and it’s *in vitro* synergy with other leukocyte attractants remained preserved. Furthermore, hrSAA1 maintained the ability to promote monocyte survival. This indicates that pure hrSAA1 retains its potential to activate FPR2, whereas TLR-mediated effects seem to be related to traces of bacterial TLR ligands in the *E. coli*-produced human rSAA1.

## Introduction

The serum amyloid A (SAA) proteins form a family that is highly conserved in a wide number of species ranging from fish to humans (1). The remarkable conservation of SAA throughout evolution points toward a rather important biological role. Humans have four distinct *SAA* genes giving rise to SAA1, SAA2, SAA3, and SAA4. SAA1 and SAA2 are upregulated during the acute phase response and are hence referred to as acute-SAA (A-SAA) (2). Expression of A-SAA primarily occurs in the liver in response to inflammatory cytokines such as interleukin-1β (IL-1β), IL-6 and tumor necrosis factor–α (TNF-α) (2). Under inflammatory conditions, SAA1 plasma levels rise exponentially (3). In contrast to mouse, human *SAA3* has long been considered a pseudogene, a fact that was recently debated (4). The role of SAA4, which is constitutively expressed, has been scarcely studied.

A considerable amount of literature has been published on the numerous biological activities attributed to SAA1, most of which are of a pro-inflammatory nature. SAA1 has been reported to upregulate the expression of various inflammatory mediators such as cell adhesion molecules, cytokines, chemokines, matrix-degrading proteases, reactive oxygen species (ROS) and pro-angiogenic molecules in several cell types including leukocytes, fibroblasts, and endothelial cells (3, 5–9). In addition, SAA1 has been described to induce the recruitment of different cell types including various leukocyte subsets (6, 10–12). Furthermore, this acute phase protein has been suggested to possess antimicrobial activity (13–16). Interestingly, SAA has been indicated as a pleiotropic molecule owing to its capacity to also induce anti-inflammatory effects (17–20).

As a multifunctional protein, SAA1 has been reported to activate various receptors. The majority of SAA1 functions has been linked to toll-like receptors (TLRs) 2 and 4 (6, 21, 22). Nevertheless, SAA1 has also been described as a ligand for additional receptors. For instance, the G protein-coupled receptor, formyl peptide receptor 2 (FPR2) has been shown to relay the direct chemotactic signal of SAA1 on FPR2-transfected HEK293 cells, neutrophils and macrophages (23–25). In addition, SAA1 was shown to synergize with CXCL8 to enhance neutrophil recruitment through activation of FPR2 (6, 26). Although the capacity to synergize with CXCL8 is retained in the C-terminal fragment of SAA1, SAA1(58–104), generated by matrix metalloproteinase-9 (MMP-9), its direct chemotactic activity is lost as a result of proteolysis by MMP-9 (27). Furthermore, SAA1 has been linked to scavenger receptor class B type I (SR-BI), receptor for advanced glycation end products (RAGE) and the purinergic receptor P2X7 (28–31).

Burgess et al. recently demonstrated that the TLR2-activating capacity of SAA1 recombinantly expressed in *Escherichia coli* (*E.coli*) is in fact due to contaminating bacterial lipoproteins (32). The analysis of such commercially available recombinant SAA1 (rSAA1) revealed the presence of multiple bacterial proteins, some of which are probable bacterial lipoproteins. Treatment of SAA1 from a bacterial source with lipoprotein lipase provoked a dose-dependent decline in the cytokine-inducing capacity of rSAA1 in peripheral blood mononuclear cells (PBMCs) and neutrophils. In line with this, SAA1 expressed in mammalian HEK293T cells did not induce inflammatory cytokine expression. Furthermore, rSAA1 from a bacterial source induced Th17 polarization whereas HEK293T-expressed SAA1 displayed no effect on these cells (32). The majority of studies performed have utilized rSAA1 that has been expressed in bacteria. Thus, it is currently unclear which functions are intrinsic to SAA1 and which are due to contaminating bacterial products. The main purpose of this study is to provide a correct understanding of the biological activities that are inherent to SAA1. Therefore, we purified commercially available rSAA1, expressed in *E. coli*, to homogeneity using reversed phase-high performance liquid chromatography (RP-HPLC). Following purification, we carried out multiple biological assays to investigate the role of homogenous rSAA1 (hrSAA1) in leukocyte survival, activation and migration, chemokine and MMP-9 induction, ROS expression and macrophage polarization. We conclude that all FPR2-mediated effects of hrSAA1 remain intact, whereas the TLR-related activities are absent.

## Materials and Methods

### Reagents

Recombinant human SAA1 (rSAA1) (300–353), CXCL8 (200–208M), IL-4 (200–204) and M-CSF (300–325) were purchased from Peprotech (Rocky Hill, NJ, United States). Lipopolysaccharide (LPS) derived from *E.coli* (0111:B4) and lipoprotein lipase (LPL) derived from *Pseudomonas* species (62335) were purchased from Sigma-Aldrich (St. Louis, MO, United States). Pam3CSK4 (11B07-MM) was purchased from InvivoGen (San Diego, CA, United States). IFN-γ (285-IF) was obtained from R&D Systems (Minneapolis, MN, United States). The FPR2 agonist MMK-1 (33) was chemically synthesized based on N-9-(fluorenyl) methoxy-carbonyl (Fmoc) chemistry using an Activo-P11 automated solid-phase peptide synthesizer (Activotec, Cambridge, United Kingdom). The peptide was purified by C18 RP-HPLC and purity was confirmed on an Amazon-SL ion trap mass spectrometer (Bruker Daltonics; Bremen, Germany). The selective FPR2 antagonist WRW_4_ was purchased from Calbiochem (San Diego, CA, United States).

### Reversed Phase High-Performance Liquid Chromatography (RP-HPLC) and Mass Spectrometry (MS)

To purify rSAA1, RP-HPLC (Higgins Analytical, Inc, Mountain View, CA, United States) coupled to MS was utilized. rSAA1 was purified on a C8 Aquapore RP-300 HPLC column (220 × 2.1 mm; PerkinElmer, Norwalk, CT, United States). The loading solvent consisted of 0.1% trifluoroacetic acid (TFA) in ultra-pure water. After loading rSAA1 onto the column, elution was achieved by a gradually increasing acetonitrile (ACN) gradient. UV absorbance was measured at 214 nm reflecting protein concentration. Following chromatographic separation, fractions containing rSAA1 were analyzed by ion trap MS. Fractions containing highly pure rSAA1 were pooled, underwent lyophilization and were reconstituted with PBS [supplemented with 1 mg/ml of human serum albumin (HSA; Belgian Red Cross, Brussels, Belgium)]. RP-HPLC-purified rSAA1 will be referred to as homogenous rSAA1 (hrSAA1) from here onward. To avoid that the activity of purified hrSAA1 would depend on batch to batch differences, the same preparation of hrSAA1 was used in chemokine and MMP-9 induction experiments, ROS production, macrophage polarization, monocyte survival, *in vitro* and *in vivo* chemotaxis, and shape change assays (*vide infra*).

### Detection of LPS and LPL Treatment

The limulus amebocyte lysate (LAL) assay was utilized to determine the endotoxin level in rSAA1 preparations before and after RP-HPLC purification. The endotoxin level in rSAA1 was measured at 2.90 EU per mg of rSAA1. Following RP-HPLC, the endotoxin level in hrSAA1 was <0.15 EU per mg of hrSAA1. The LAL assay was carried out using a specific kit as per the manufacturer’s instructions (BioMérieux; Marcy-l’Étoile, France). To deactivate bacterial lipoproteins in the rSAA1 preparation, rSAA1 was pre-incubated with LPL for a period of 4 h at 37°C in CD14^+^ monocyte culture medium prior to cell stimulation.

### Sodium Dodecyl Sulfate-Polyacrylamide Gel Electrophoresis (SDS-PAGE)

hrSAA1 and rSAA1 were diluted in reducing loading buffer (10% beta mercaptoethanol, 0.02% bromophenol blue, 8% SDS, 40% glycerol in 0.25 M tris pH 6.8) and heated at 95°C for 5 min. Afterward, the samples were loaded onto a precast 16% tris-glycine gel (Invitrogen, Carlsbad, CA, United States) and run at 200 V in running buffer (192 mM glycine, 0.1% SDS in 25 mM tris pH 8.6). Following electrophoresis, the gel was stained using a silver stain kit as per the manufacturer’s instructions (Invitrogen).

### Signal Transduction Assay

FPR1- and FPR2-transfected HEK293 cells were kindly provided by Prof. J.M. Wang (NCI, Frederick, United States). Changes in intracellular calcium concentration were measured by fluorescence spectrometry as previously described (34). In brief, HEK293 cells were loaded with the ratiometric fluorescent dye Fura-2/AM (Invitrogen) and incubated for a period of 0.5 h at 37°C. Afterward, the cells were washed and resuspended in HBSS containing 1 mM Ca^2+^ and Mg^2+^ (Gibco, Thermo Fischer Scientific, Waltham, MA, United States), 0.1% fetal calf serum (FCS, Invitrogen), 10 mM HEPES and pH 7.4, at a concentration of 1.5 × 10^6^ cells/ml. Fura-2 fluorescence was measured at 510 nm upon excitation at 340 and 380 nm.

### Human Monocyte Isolation and Activation

Human CD14^+^ monocytes were isolated from 1-day-old buffy coats, obtained from healthy donors (Belgian Red Cross, Mechelen, Belgium), via density gradient centrifugation and positive selection (MACS, Miltenyi Biotec, Bergisch Gladbach, Germany) as previously described (26). CD14^+^ monocytes were seeded in 48-well plates (2 × 10^6^ cells/ml, 450 μl/well) in RPMI-1640 medium (Lonza, Basel, Switzerland) supplemented with 1 mg/ml HSA. Monocytes were stimulated for 24 h at 37°C and 5% CO_2_. Cell supernatants were collected and stored at −20°C until chemokine and MMP-9 quantification.

### Human Neutrophilic Granulocytes Isolation and Activation, Migration, and Cell Shape Assays

Human neutrophils were isolated from fresh blood, obtained from healthy donors via density gradient centrifugation as previously described (6). Neutrophil migration was determined in a 48-well Boyden microchamber assay (Neuro Probe, Gaithersburg, MD, United States). Chemoattractants were added in triplicate to the lower wells of the microchamber. Boyden buffer (HBSS supplemented with 1 mg/ml of HSA) served as negative control. Neutrophils (1 × 10^6^ cells/ml, 50 μl/well) diluted in Boyden buffer were added to the upper compartment, which was separated from the lower compartment using a polyvinylpyrrolidone-free membrane (5 μm pore size; GE water & process Technologies, Manchester, United Kingdom). After an incubation period of 45 min at 37°C and 5% CO_2_, the membrane was fixed and stained with Hemacolor solutions (Merck, Darmstadt, Germany). The migrated cells adhering to the bottom surface of the membrane were counted microscopically in 10 high power fields/well. The chemotactic potency was expressed in terms of the chemotactic index (CI). The CI was calculated by dividing the average number of migrated cells in response to chemoattractants by the average number of spontaneously migrated cells.

Shape change assays were carried out to determine the morphological changes that occur when neutrophils are stimulated with chemoattractants in suspension. Different concentrations of inducers (50 μl) were added to a flat-bottomed 96-well plate. The stimuli and neutrophils were diluted in pre-warmed (37°C) shape change buffer (HBSS, supplemented with 10 mM HEPES), which also served as the negative control. Neutrophils (50 μl/well) were then added to the plate at a concentration of 0.6 × 10^6^ cells/ml. Following 3 min of stimulation, neutrophils were fixed with 100 μl of 4% formaldehyde in shape change buffer. One hundred cells per condition were counted microscopically and categorized as either active (blebbed and elongated cells) or resting/not activated (round). Synergy was defined as a response to the combination of two chemoattractants that exceeded the sum of the responses obtained for the individual chemoattractants.

### CXCL8 and CCL3 Enzyme-Linked Immunosorbent Assay (ELISA)

Quantification of CXCL8 in monocyte supernatants was done by ELISA. The human CXCL8 ELISA was developed in our laboratory using monoclonal mouse anti-human CXCL8 (MAB208) and polyclonal goat anti-human CXCL8 (BAF208) antibodies from R&D Systems (6). Human CCL3 was measured with a specific ELISA Duoset kit as per the manufacturer’s instructions (R&D Systems).

### MMP-9 Zymography

MMP-9 release by activated CD14^+^ monocytes was quantified as described by Vandooren *et al.* (35). Monocyte cell supernatants, diluted in a non-reducing loading buffer (0.02% bromophenol blue, 8% SDS, 40% glycerol in 0.25 M tris pH 6.8), were loaded onto 7.5% polyacrylamide gels containing 0.1% gelatin and run at 25 mAmp. Following electrophoresis, gels were washed with 2.5% triton-X 100 in ultrapure water. Afterward, the gels were incubated overnight in incubation buffer (10 mM CaCl_2_ in 50 mM tris pH 7.4) for the development of enzyme activity. The gels were then stained with InstantBlue^TM^ protein stain (Expedeon, Heidelberg, Germany) as per the manufacturer’s instructions and destained for 1 h in destaining solution (30% methanol and 10% acetic acid in ultrapure water). MMP-9 gelatinase activity was observed as unstained bands on a blue background. The obtained signals were quantified by computerized image analysis using ImageJ software.

### Cellular ROS Assay

CD14^+^ monocytes (2 × 10^6^ cell/ml, 200 μl) were incubated with stimuli (100 μl) in pre-warmed RPMI-1640 medium (Lonza), supplemented with 2% FCS for a period of 1 h at 37°C and 5% CO_2_. ROS generation was detected using 50 μM (in 100 μl) of 2, 7-dichlorodihydrofluorescein diacetate (DCFH-DA; Sigma-Aldrich) which was added for 20 min at 37°C following cell stimulation. The cells were placed on ice for 10 min to stop the reaction and subsequently washed with HBSS buffer. Afterward, the cells were fixed using 0.8% formaldehyde. Fluorescence intensity was measured using an LSRFortessa X-20 cell analyzer (BD Biosciences, Heidelberg, Germany) at 488 nm. Data were analyzed using FlowJo software (Tree Star, Ashland, OR, United States).

### Macrophage Differentiation

CD14^+^ monocytes were suspended at a concentration of 2 × 10^6^ cells/ml in RPMI1640 medium, supplemented with 10% FCS and 50 μg/ml gentamycin, and cultured in 6-well plates (2 ml/well). To induce the differentiation of human monocytes into macrophages, M-CSF (100 ng/ml) was added on day 0. The stimuli were added on day 4. On day 6 of culture, macrophages were collected from the 6-well plates and processed for analysis of surface molecules via flow cytometry (*vide supra*). To exclude dead cells from the analysis, cells were incubated in Zombie Aqua viability dye (BioLegend, San Diego, CA, United States) for 15 min at room temperature. To block the Fc receptors, the cells were washed and incubated for 10 min at 4°C with FACS buffer (PBS + 2% FCS + 1 mM EDTA). Afterward, the cells were stained with the mouse anti-human antibodies mentioned hereafter (0.5 h at 4°C) and analyzed by flow cytometry (*vide supra*). Fluorescein Isothiocyanate (FITC)-labeled anti-TLR4 antibody (clone HTA125) was obtained from InvivoGen. FITC-labeled anti-HLA-DR antibody (clone LN3) and phycoerythrin (PE)-labeled anti-CD163 antibody (clone GHI/61) were obtained from eBioscience (San Diego, CA, United States). PE-labeled anti-IL-1RI (clone 150503) and anti-CCR7 (catalog number FAB269P) antibodies were obtained from R&D Systems. FITC-labeled anti-DC-SIGN (clone DCN46), PE-labeled anti-CD80 (clone L307.4), PE-labeled anti-CD86 (clone 2331), Brilliant Violet 421 (BV421)-labeled anti-TLR2 (clone 1167) and allophycocyanin (APC)-labeled anti-CD14 (clone M5E2) antibodies were obtained from BD Biosciences. The degree of polarization is expressed as percentage change of marker expression compared to control M-CSF-differentiated macrophages.

### Monocyte Survival

Peripheral blood mononuclear cells were suspended in RPMI1640 supplemented with 1% FCS and seeded at a concentration of 1.5 × 10^6^ cells/ml (100 μl/well) in a 96-well plate. Monocytes were allowed to adhere for 2 h at 37°C and then washed with PBS. Following a stimulation period of 24 h, monocyte survival was assessed using the ATPlite^TM^ luminescence assay system kit as per the manufacturer’s instructions (PerkinElmer). Results are expressed as percent of survival in comparison to buffer-stimulated monocytes (100%).

### Actin Polymerization

To monitor changes in the cytoskeleton in response to stimuli, neutrophils were seeded in a U bottom 96-well plate at 1.5 × 10^6^ cells/ml (70 μl/well) in pre-warmed (37°C) RPMI1640 + 0.5% HSA. After stimulation for 30 sec, cells were placed on ice, fixed and permeabilized using the BD Cytofix/Cytoperm^TM^ kit (BD Biosciences) as per the manufacturer’s instructions. Subsequently, cells were incubated (20 min on ice) with Alexa Fluor^TM^ 555 Phalloidin (20 U/ml, Invitrogen), a dye which selectively stains F-actin. After washing, the cellular F-actin content was quantified by flow cytometry (*vide supra*). Results were expressed as relative mean fluorescence intensity (MFI), i.e. compared to the MFI of buffer-stimulated cells (100%).

### Intra-Articular (i.a.) Knee Injections

The *in vivo* neutrophil chemotactic potential of hrSAA1 was determined in C57BL/6J male mice (Centro de Bioterismo of the Universidade Federal de Minas Gerais) via i.a. injections. The mice were first anesthetized through intraperitoneal (i.p.) injection of a mixture of 3.75% (w/v) of ketamine (Syntec, Santano de Parnaíba, Brazil) and 0.25% (w/v) of xylazine (Syntec) diluted in PBS. Afterward, the mice were injected i.a. with 10 μl of stimulus in one joint and, as a control, the other joint was injected with 10 μl of 0.9% sodium chloride. After 3 h, mice were sacrificed by a subcutaneous injection of a ketamine/xylazine overdose. Cells from the joint were collected and cytospins were prepared for differential cell counts. After drying, cells on the glass slides were stained with Panoptic solutions (Laborclin, PR, Brazil). The slides were microscopically counted (500× magnification) independently by 2 individuals. All procedures were approved by the animal ethics committee of the Federal University of Minas Gerais (295/2018).

### Statistical Analysis

The data were first analyzed using the Kruskal–Wallis test for comparison of multiple groups. Afterward, the Mann-Whitney U-test or the Wilcoxon signed-rank test were utilized to perform pairwise comparisons. Statistical analysis was performed using GraphPad software (GraphPad Software Inc. La Jolla, United States). Unless indicated otherwise, results are expressed as mean + SEM. An alpha of 0.05 was used as the cutoff for significance.

## Results

### Purification of rSAA1 to Homogeneity

**FIGURE 1:**
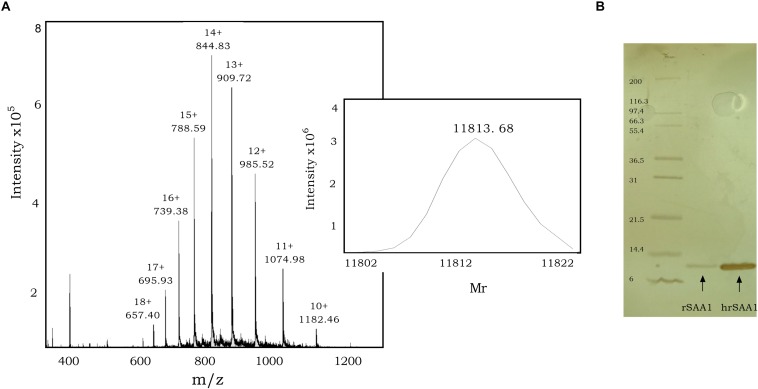
Purification of rSAA1 using RP-HPLC to homogeneity. **(A)** Relative molecular mass determination of RP-HPLC-purified homogenous rSAA1 (hrSAA1) by mass spectrometry. The averaged mass spectrum of the pooled hrSAA1 fractions is shown with the ion intensities, the number of charges and the corresponding mass over charge ratio (m/z) for multiple charged ions. The experimentally determined deconvoluted mass spectrum of uncharged hrSAA1, as calculated by the Bruker deconvolution software, is shown as an insert at the right of the mass spectrum. **(B)** Homogenous rSAA1 (hrSAA1; 200 ng) was analyzed in parallel to rSAA1 (20 ng) using SDS–PAGE and silver staining. The molecular mass of the standard marker proteins is indicated in kilodalton.

**FIGURE 2:**
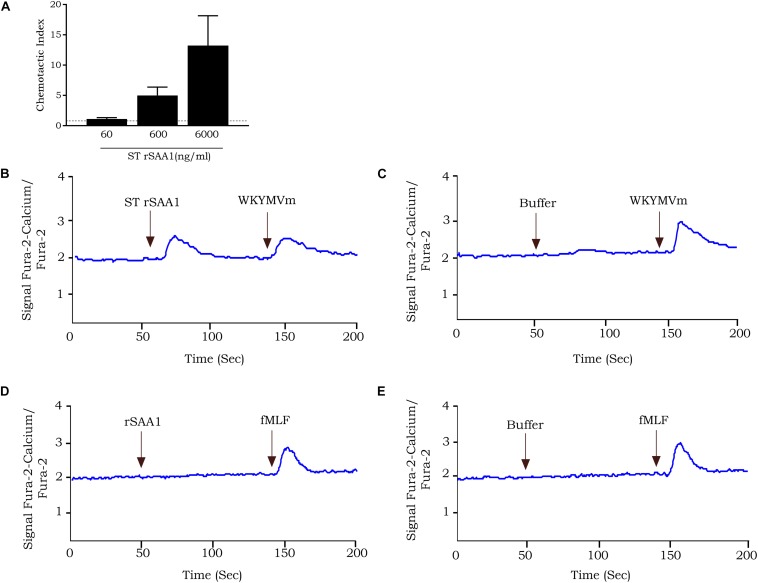
rSAA1 treated with RP-HPLC elution solvents retains its biological activity. **(A)** The chemotactic activity of rSAA1 treated with 50% acetonitrile and 0.1% trifluoroacetic acid (ST rSAA1) was evaluated on human neutrophils in the Boyden microchamber assay. The chemotactic potencies are expressed as mean chemotactic index + SEM derived from three independent experiments. Control migration is indicated by a dashed line (—). **(B)** FPR2-transfected HEK293 cells were stimulated with rSAA1 (6000 ng/ml, 500 nM) treated with 50% acetonitrile and 0.1% trifluoroacetic acid. Afterward, the cells were stimulated with WKYMVm (10 ng/ml, 12 nM) as control. **(C)** FPR2-transfected HEK293 cells were stimulated with WKYMVm (10 ng/ml, 12 nM). **(D)** FPR1-transfected HEK293 cells were stimulated with rSAA1 (6000 ng/ml, 500 nM). Afterward, the cells were stimulated with fMLF (10^–10^ M) as control. **(E)** FPR1-transfected HEK293 cells were stimulated with fMLF (10^–10^ M). **(B–E)** Changes in intracellular calcium levels were monitored by spectrophotometry. Results are presented as the ratio of emission of calcium-bound fura over calcium-free fura. One representative experiment out of two independent experiments is shown.

To further eliminate bacterial components, such as lipoproteins, lipopolysaccharides, and formyl peptides from commercial rSAA1 expressed in *E.coli*, we performed RP-HPLC. rSAA1 eluted from the C8 column between 50.0% and 58.0% ACN in 0.1% TFA. Following RP-HPLC purification, mass spectrometry was used to assess the purity and confirm the presence of homogeneous (h) rSAA1. Figure 1A shows the mass spectrum obtained following rSAA1 purification with ions containing 10–18 charges. The insert in the figure shows the deconvoluted relative molecular mass of hrSAA1 (11813.68) which aligns with the theoretical relative molecular weight of rSAA1 (11813.78). To further assess the purity of the preparation, hrSAA1 was analyzed by SDS-PAGE, which displayed no major contaminants (Figure 1B). In order to rule out the possibility that exposure to harsh solvents (50% ACN in 0.1% TFA) would alter the biological activity of hrSAA1, rSAA1 was incubated in 50% ACN in 0.1% TFA for a period of 1 h followed by lyophilization and reconstitution. Solvent-treated rSAA1 (ST rSAA1) retained biological function, including neutrophil chemotactic activity (Figure 2A). In addition, ST-rSAA1 induced calcium mobilization in FPR2-transfected HEK293 cells (Figure 2B) but did not significantly desensitize the calcium response induced by the FPR2 agonist WKYMVm (Figure 2C). Furthermore, ST rSAA1 retained the chemokine-inductive capacity in CD14^+^ monocytes (data not shown). hrSAA1 was then utilized in multiple biological assays in parallel to rSAA1.

### Absence of Formyl Peptide Contamination and Separation of Bacterial Lipoprotein From rSAA1 by RP-HPLC

Contamination with formyl peptides was excluded, as neither rSAA1 (Figure 2D), nor hrSAA1 (data not shown) induced calcium signaling in FPR1-transfected HEK293 cells. Furthermore, rSAA1 failed to desensitize calcium signaling in response to fMLF in FPR1-transfected cells, thus further confirming the lack of formyl peptides in the preparation (Figures 2D,E). Currently, there exists no method to detect and quantify low levels of contaminating bacterial lipoproteins. Therefore, to confirm that bacterial lipoproteins present in rSAA1 do not co-elute with rSAA1 during RP-HPLC purification, Pam3CSK4, a synthetic bacterial lipoprotein variant, was loaded onto a C8 Aquapore RP-300 HPLC column. Elution was carried out in a similar manner to that of rSAA1. The elution of Pam3CSK4 was achieved at a solvent composition of 76.6–100.0% ACN in 0.1% TFA. As previously mentioned, rSAA1 eluted at a solvent composition of 50.0–58.0% ACN in 0.1% TFA, indicating that rSAA1 does not co-elute with contaminating bacterial lipoproteins under the described conditions of RP-HPLC (data not shown).

### hrSAA1 Fails to Induce Chemokine Production in CD14^+^ Monocytes

**FIGURE 3:**
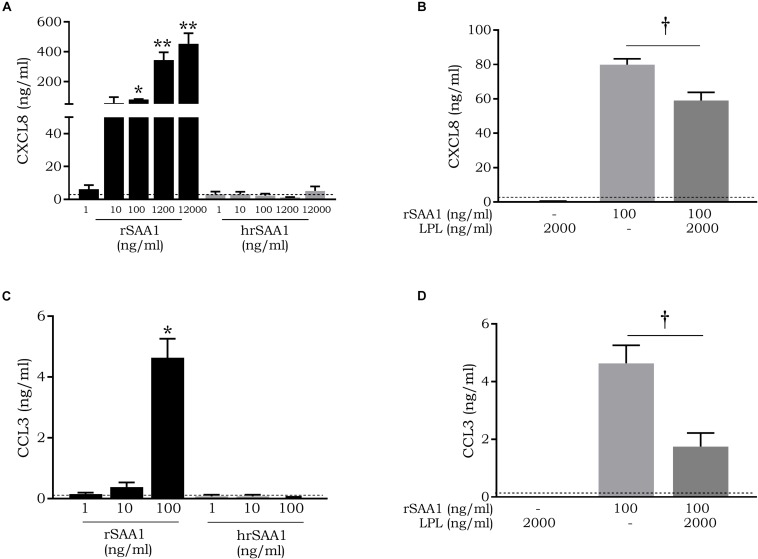
hrSAA1 does not induce chemokine expression in CD14^+^ monocytes and treatment of rSAA1 with LPL diminishes activity. **(A,C)** Freshly isolated monocytes were induced with homogenous rSAA1 (hrSAA1) (1–12000 ng/ml, 0.08–1000 nM) in parallel with rSAA1 (1–12000 ng/ml, 0.08–1000 nM). **(B,D)** rSAA1 (100 ng/ml, 8 nM) was pre-incubated with LPL (2000 ng/ml) for 4 h prior to stimulation of monocytes. Following an incubation period of 24 h, cell supernatants were collected and CXCL8 and CCL3 expression was determined via specific ELISAs. Expression in unstimulated cells is indicated by a dashed line (—). Results are represented as the mean chemokine concentration + SEM and are derived from four to six independent experiments. Significant upregulation in comparison to control is indicated by asterisks (Mann-Whitney *U*-test; *p-value < 0.05, **p-value < 0.01). Significant reduction in inductive capacity following LPL treatment is indicated by a dagger (Mann-Whitney *U*-test; †*p*-value < 0.05).

rSAA1 has been reported to induce the expression of chemokines including CCL2, CCL3, and CXCL8 in monocytes and dendritic cells (11). As such, monocytes were stimulated with hrSAA1 in parallel to rSAA1 to determine whether rSAA1 retains the capacity to induce chemokines following purification. Following a stimulation period of 24 h, CCL3 and CXCL8 levels were determined in the cell supernatants. In comparison to rSAA1 (CCL3 4.6 ± 0.6 ng/ml and CXCL8 79.9 ± 3.4 ng/ml), hrSAA1 did not induce significant CCL3 nor CXCL8 expression in monocytes at 100 ng/ml (CCL3 was undetectable and CXCL8 2.3 ± 1.2 ng/ml) (Figures 3A,C). Furthermore, as shown in Figures 3B,D, treatment of rSAA1 (100 ng/ml) with LPL (2000 ng/ml) reduced CCL3 and CXCL8 expression in monocytes by 62.3 (*p*-value 0.0286) and 26.2% (*p*-value 0.0286), respectively, confirming the results of Burgess *et al.* (32). SAA shows a diverse set of functions which are conveyed at a wide range of varying concentrations from as low as 12.5 ng/ml to 50000 ng/ml (2). To confirm that hrSAA1 does not induce chemokine expression due to the lack of inherent capacity and not due to too low concentrations used (1–100 ng/ml), hrSAA1 was also added at higher concentrations (1200 ng/ml and 12000 ng/ml) to CD14^+^ monocytes, but failed to induce CXCL8 production (Figure 3A).

### hrSAA1 Fails to Induce MMP-9 Release by CD14^+^ Monocytes

**FIGURE 4:**
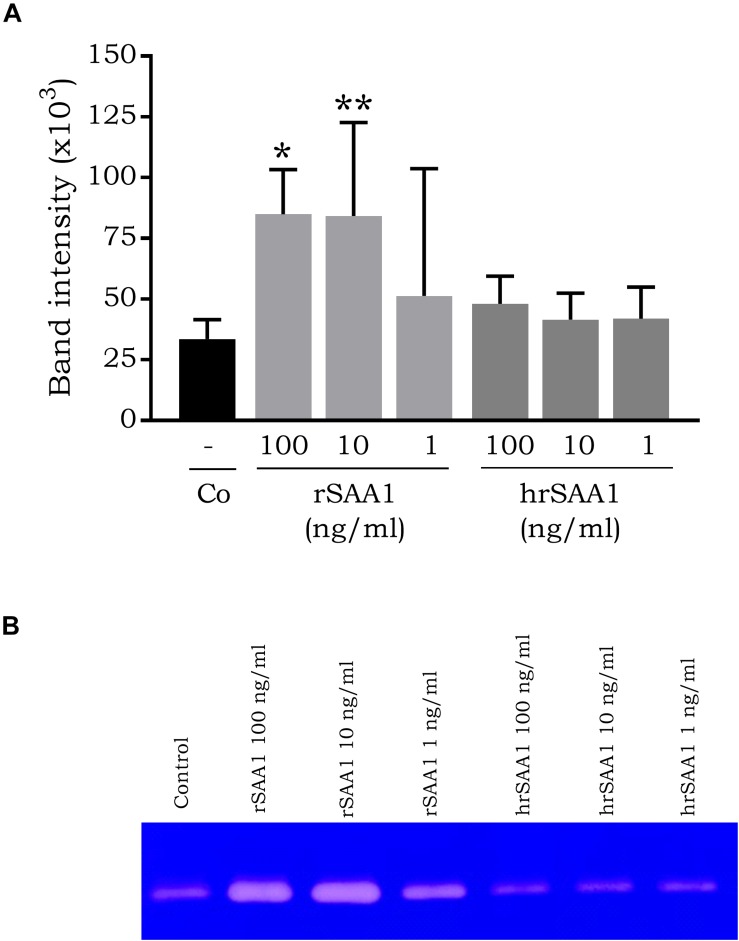
hrSAA1 does not induce MMP-9 expression in CD14^+^ monocytes. Freshly isolated monocytes were induced with homogenous rSAA1 (hrSAA1) (1–100 ng/ml, 0.08–8 nM) in parallel with rSAA1 (1–100 ng/ml, 0.08–8 nM). Following an incubation period of 24 h, cell supernatants were collected and MMP-9 expression was determined via zymography. **(A)** Results are represented as the mean band intensity (×10^3^) + SEM and are derived from four to six independent experiments. Significant upregulation in comparison to control is indicated by asterisks (Mann-Whitney *U*-test; *p-value < 0.05, **p-value < 0.01). **(B)** One representative zymography is shown.

Connolly *et al*. demonstrated the expression of MMP-1, MMP-2, MMP-3, MMP-9, and MMP-13 by fibroblast-like synoviocytes in response to rSAA1 stimulation (9). In addition, rSAA1 was also described to induce MMP-10 upregulation in human umbilical vein endothelial cells (36). In order to determine whether the MMP-inducing capacity of rSAA1 is endogenous to SAA1, monocytes were stimulated with rSAA1 and hrSAA1. Following a 24 h stimulation period, cell supernatants were collected and MMP-9 activity was determined using zymography. Significant expression of MMP-9 was not observed in response to hrSAA1 stimulation (band intensity 48.1 ± 11.4 × 10^3^ versus control 33.5 ± 8.0 × 10^3^) (Figure 4). On the other hand, rSAA1 induced significant MMP-9 expression at 100 ng/ml (band intensity 84.9 ± 1.8 × 10^3^).

### hrSAA1 Fails to Induce ROS Production in CD14^+^ Monocytes

**FIGURE 5:**
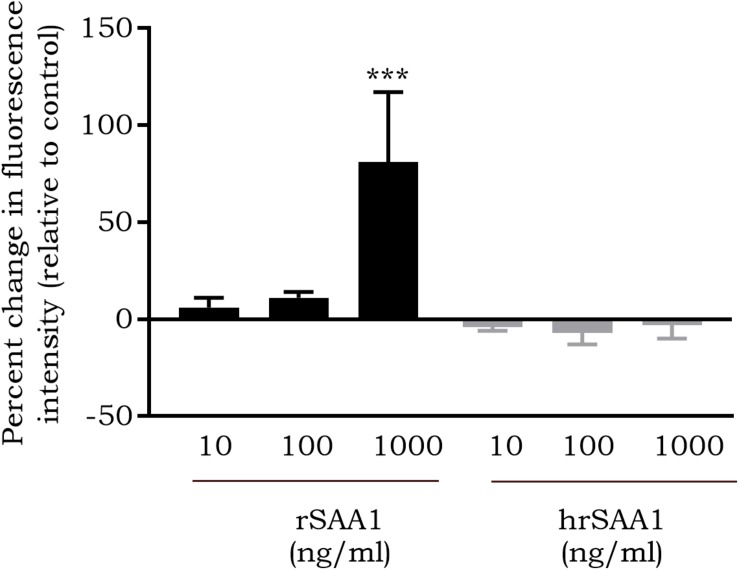
hrSAA1 does not induce ROS production in CD14^+^ monocytes. Freshly isolated monocytes were stimulated with homogenous rSAA1 (hrSAA1) (10–1000 ng/ml, 0.8–80 nM) in parallel with rSAA1 (10–1000 ng/ml, 0.8–80 nM). Following an incubation period of 1 h, staining with 2′,7′-dichlorofluorescein diacetate (50 μM) was carried out and flow cytometry was used to quantify ROS production. Results are presented as the mean percent change in fluorescence intensity in comparison to buffer-treated cells + SEM (i.e., 0% is equivalent to the same fluorescence intensity as obtained for buffer-treated cells) and are derived from four to seven independent experiments. Significant upregulation in comparison to control is indicated by asterisks (Mann-Whitney *U*-test; ***p-value < 0.001).

In response to rSAA1 stimulation, ROS expression by several cell types, such as glioma cells, fibroblasts and neutrophils, has been reported (37–39). In addition, rSAA1 has been described to activate NADPH-oxidase, thereby contributing to the production of superoxide (40). Furthermore, rSAA1 has been shown to prime neutrophils to enhance ROS production in response to zymosan (39). To determine whether SAA1 possesses the capacity to induce ROS expression, monocytes were stimulated for 1 h with hrSAA1 in parallel with rSAA1 and ROS production was measured through DCFH-DA staining. In line with previously published results, rSAA1 induced a dose-dependent increase in ROS expression. At 1000 ng/ml, rSAA1 induced an 81 ± 36% increase in ROS expression in comparison to control (*p*-value 0.0006, Figure 5). In contrast, hrSAA1 did not induce notable ROS expression in monocytes (−3 ± 7% change at 1000 ng/ml).

### hrSAA1 Lacks Macrophage Polarizing Capacity

**FIGURE 6:**
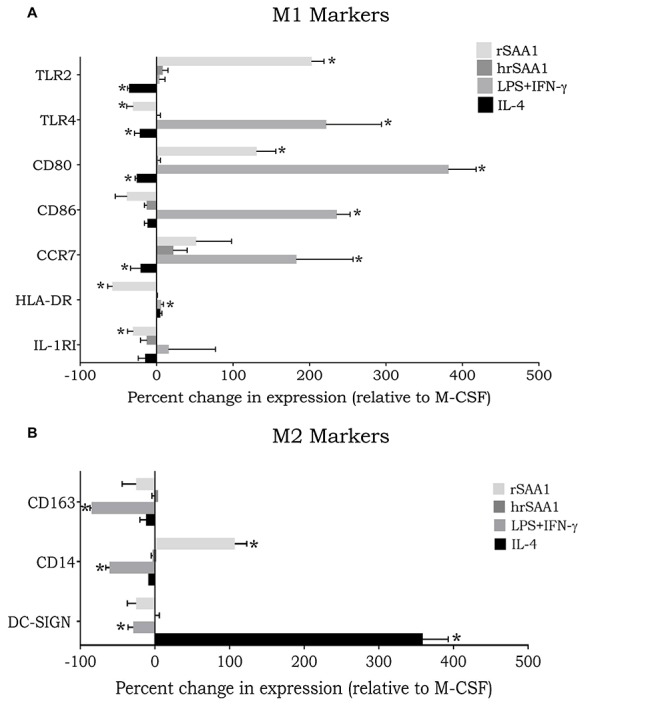
hrSAA1 does not mediate macrophage polarization. Freshly isolated monocytes were treated with M-CSF (100 ng/ml, 2.7 nM) on day 0 to induce macrophage differentiation. On day 4 cells were treated with IFN-γ (25 ng/ml, 1.5 nM) plus LPS (100 ng/ml), IL-4 (20 ng/ml, 1.3 nM), rSAA1 (100 ng/ml, 8 nM), or homogenous rSAA1 (hrSAA1) (100 ng/ml, 8 nM) to promote macrophage polarization. On day 6 of culture, macrophages were collected and analyzed for expression of **(A)** M1 markers and **(B)** M2 markers using flow cytometry. Results are presented as the mean percent change in expression compared to M-CSF-treated cells + SEM (i.e., 0% is equivalent to the same expression level as M-CSF-treated cells) and are derived from seven independent experiments. Significant upregulation in comparison to control is indicated by asterisks (Wilcoxon signed-rank test; *p-value < 0.05).

Previous studies have explored the capacity of rSAA1 to regulate macrophage polarization. Indeed, Li et al. have observed the polarization of U937 cells into M2b macrophages in response to SAA stimulation (41). In line with this, Sun et al. demonstrated M2 polarization of human CD14^+^ monocytes in response to rSAA (42). On the contrary, Anthony et al. demonstrated a mixed M1/M2 phenotype following stimulation of blood monocytes with rSAA1 (43). To compare the phenotype of hrSAA1-stimulated to rSAA1-stimulated macrophages, the expression of several surface markers (relative to M-CSF-differentiated macrophages) was determined. IFN-γ (100 ng/ml) combined with LPS (25 ng/ml) or IL-4 (20 ng/ml) were added as controls to skew toward the M1 or M2 phenotype, respectively. As anticipated, LPS combined with IFN-γ upregulated a number of M1 markers including CCR7 (183 ± 74%), CD80 (382 ± 36%), CD86 (236 ± 17%), and TLR4 (222 ± 72%) (Figure 6A). Similarly, IL-4 upregulated the M2 marker DC-SIGN (359 ± 34%). rSAA1 (100 ng/ml) induced the downregulation of a number of M1 markers including IL-1 receptor type I (IL-1RI) (−31 ± 8%), HLA-DR (−58 ± 6%) and TLR4 (−31 ± 8%), whereas it also upregulated a number of M1 markers including CD80 (131 ± 25%) and TLR2 (203 ± 16%). Furthermore, rSAA1 increased the CD14 expression level (107 ± 16%) (Figure 6B). In sharp contrast, hrSAA1 (100 ng/ml) did not exert any effect on the expression of M1 or M2 macrophage markers.

### hrSAA1 Promotes Monocyte Survival

**FIGURE 7:**
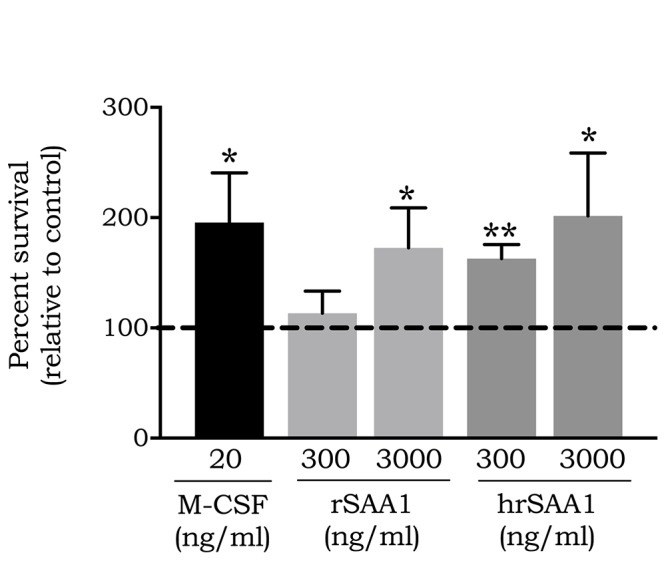
hrSAA1 promotes the survival of monocytes. Freshly isolated monocytes were treated with hrSAA1 (300 or 3000 ng/ml, 25 or 250 nM), rSAA1 (300 or 3000 ng/ml, 25 or 250 nM), M-CSF (20 ng/ml, 0.54 nM) or left untreated (control) for a period of 24 h. Following stimulation, ATP levels were detected using firefly luciferase luminescence. Control survival is indicated by a dashed line (—). Results are presented as the mean percent of survival in comparison to control + SEM and are derived from five independent experiments. Significant survival in comparison to control is indicated by asterisks (Mann-Whitney *U*-test; *p-value < 0.05, **p-value < 0.01).

A number of studies have demonstrated the capacity of rSAA1 to promote the survival of leukocytes namely neutrophils via the activation of FPR2 (44, 45). More interestingly, also plasma-derived SAA, which lacks the inflammatory capacity displayed by its recombinant counterpart, was shown to promote neutrophil survival (46). To determine whether rSAA1 free from bacterial contaminants can promote leukocyte survival, monocytes were treated during 24 h with hrSAA1 (300 and 3000 ng/ml) in parallel with rSAA1 (300 and 3000 ng/ml) and M-CSF (20 ng/ml). Cell viability was assessed through the quantification of ATP in the cell lysates. Following a 24 h stimulation period, 300 and 3000 ng/ml of hrSAA1 (162.8 ± 12.7% and 201.5 ± 57%, *p*-value 0.0094 and 0.0136, respectively) promoted notable monocyte survival in comparison to medium treatment (100.0 ± 11.1%; Figure 7). Albeit slightly less effective than hrSAA1, rSAA1 at 3000 ng/ml also induced statistically significant monocyte survival (172.5 ± 36.3%, *p*-value 0.0136). Furthermore, M-CSF, an established monocyte survival factor, enhanced monocyte survival at 20 ng/ml (195.5 ± 45.2%, *p*-value 0.0268).

### hrSAA1 Retains Its Leukocyte Recruiting Capacity *in vivo*

**FIGURE 8:**
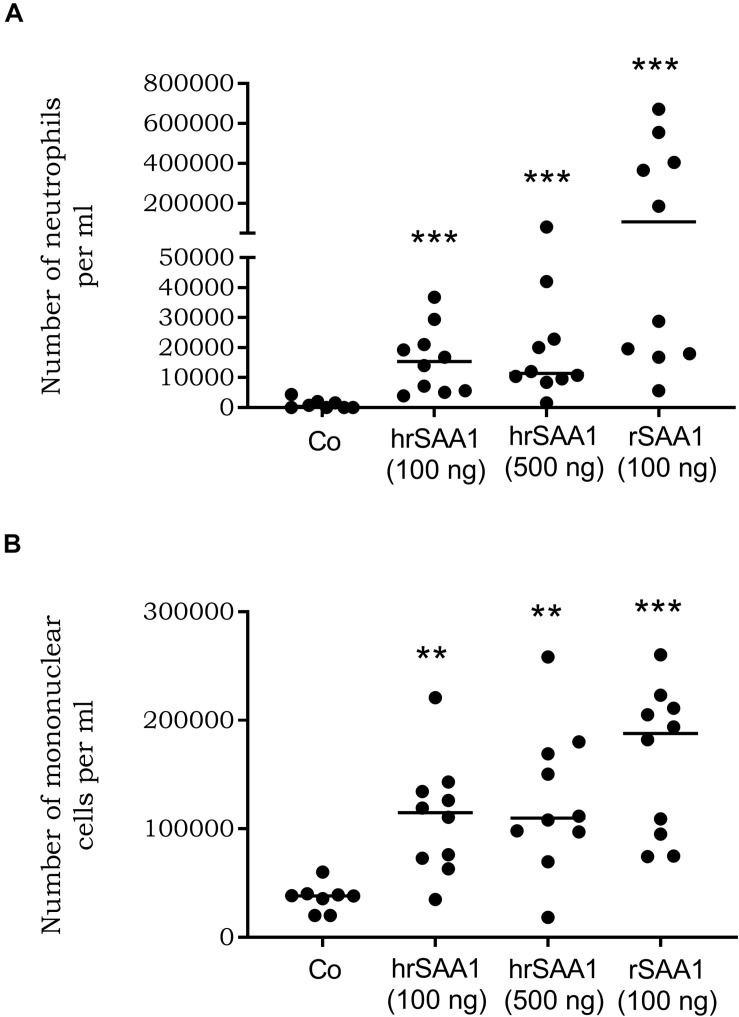
hrSAA1 induces *in vivo* recruitment of neutrophils and mononuclear cells. C57BL/6J male mice (8–10 mice per group) were intra-articularly injected with homogenous rSAA1 (hrSAA1; 100 or 500 ng/10 μl), rSAA1 (100 ng/10 μl) or 0.9% sodium chloride (control; 10 μl). Following an incubation period of 3 h, cells from the joint were collected and cytospins were prepared. Each dot represents one mouse and the horizontal line indicates the median number of recruited **(A)** neutrophils and **(B)** mononuclear cells per ml. Data are derived from two independent experiments. Significant leukocyte recruitment in comparison to control is indicated by asterisks (Mann-Whitney *U*-test; **p-value < 0.01, ***p-value < 0.001).

Various studies, utilizing recombinantly expressed SAA1, have demonstrated the chemotactic potential of SAA1. rSAA1 has been described as an *in vitro* chemoattractant for a wide range of cells including endothelial cells, fibroblasts, immature dendritic cells, mast cells, monocytes, neutrophils, smooth muscle cells, and T cells (6, 11, 12, 47–50). In addition, the *in vivo* chemotactic activity of rSAA1 has been well documented (10). To verify whether pure hrSAA1 retains its chemoattractant capacity, C57BL/6J mice were i.a. injected with hrSAA1 (100 ng/10 μl or 500 ng/10 μl) in parallel with rSAA1 (100 ng/10 μl). Injection of hrSAA1 (500 ng/10 μl i.a.) induced significant recruitment (28-fold increase, Figure 8) of neutrophils in comparison to control [11400 versus 400 cells per ml (median values); *p*-value 0.0002]. Furthermore, hrSAA1 (500 ng/10 μl) induced the recruitment of mononuclear cells into the joint cavity (109800 cells per ml) which is evidenced by an approximate threefold increase in the number of mononuclear cells compared to control (38200 cells per ml; *p*-value 0.003). In a similar manner, rSAA1 induced the recruitment of neutrophils and mononuclear cells into the joint cavity (107500 and 187900 cells per ml, respectively). However, rSAA1 displayed a higher potency which could be attributed to contaminating bacterial products (*p*-value < 0.05; 100 ng/10 μl of rSAA1 versus both 100 and 500 ng/10 μl of hrSAA1).

### hrSAA1 Synergizes With CXCL8 to Activate and Chemoattract Neutrophils *in vitro* via FPR2 Activation

**FIGURE 9:**
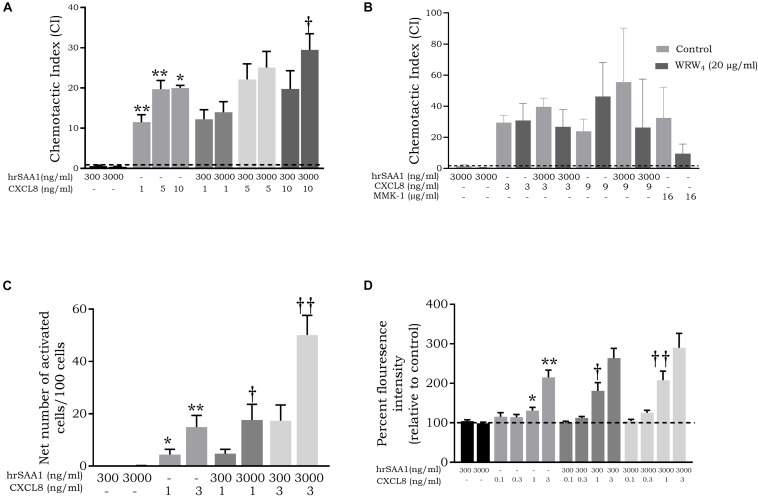
hrSAA1 retains the capacity to synergize with CXCL8 in the *in vitro* attraction and activation of neutrophils. **(A)** Homogenous rSAA1 (hrSAA1, 300 or 3000 ng/ml, 25 or 250 nM) and CXCL8 (1–10 ng/ml, 0.12–1.2 nM) were added to the lower compartment of a Boyden microchamber either alone or in combination. Freshly isolated neutrophils were added to the upper compartment and allowed to migrate for 45 min. The chemotactic potencies are expressed as mean chemotactic index (+SEM) derived from six independent experiments. Control recruitment is indicated by a dashed line (—). **(B)** MMK-1 (16 μg/ml, 9930 nM), hrSAA1 (3000 ng/ml, 250 nM), CXCL8 (3 or 9 ng/ml, 0.36 or 1.07 nM) or a combination of hrSAA1 and CXCL8 were added to the lower compartment of a Boyden microchamber. Freshly isolated neutrophils in the presence or absence of the FPR2 antagonist WRW_4_ (20 μg/ml, 18110 nM) were added to the upper compartment and allowed to migrate for 45 min. The chemotactic potencies are expressed as mean chemotactic index + SEM and are derived from three independent experiments. Control recruitment is indicated by a dashed line (—). **(C)** Neutrophils were stimulated (3 min) with homogenous hrSAA1 (300 or 3000 ng/ml, 25 or 250 nM), CXCL8 (1 or 3 ng/ml, 0.12 or 0.36 nM) or a combination of hrSAA1 and CXCL8. Data from six independent experiments are expressed as the net percentage of activated cells (blebbed and elongated cells) (+SEM). **(D)** Freshly isolated neutrophils were stimulated for 30 s with different concentrations of hrSAA1, CXCL8 or a combination of hrSAA1 and CXCL8. Following stimulation, cells were stained with Alexa Fluor 555 Phalloidin. Control actin polymerization is indicated by a dashed line (—). Results are presented as the mean percent florescence intensity relative to buffer-stimulated cells (+SEM) and are derived from six independent experiments. Significant neutrophil recruitment/activation in comparison to control is indicated by asterisks (Mann-Whitney *U*-test; *p-value < 0.05, **p-value < 0.01). Significant synergy between hrSAA1 and CXCL8 in neutrophil activation/recruitment is indicated by daggers (Mann-Whitney *U*-test; †*p*-value < 0.05, ††*p*-value < 0.01).

De Buck et al. have previously reported a synergistic effect between SAA1 and CXCL8 in the recruitment of neutrophils (6, 26). To this end, we carried out chemotaxis experiments on neutrophils using combinations of CXCL8 and hrSAA1. hrSAA1 retained the capacity to synergize with CXCL8 in neutrophil recruitment which was evidenced by the enhanced chemotactic indices (CI) observed with the combination of hrSAA1 and CXCL8 (CI = 0.4 ± 0.1 for 3000 ng/ml hrSAA1, CI = 20.0 ± 4.0 for 10 ng/ml CXCL8 versus CI = 29.5 ± 4.0 for 3000 ng/ml hrSAA1 + 10 ng/ml CXCL8, *p*-value 0.0286; Figure 9A). Moreover, in line with previous studies reporting the usage of FPR2 by SAA1 during synergy with CXCL8 during neutrophil recruitment (6, 26, 27), we observed the inhibition of the synergistic effect between hrSAA1 and CXCL8 in response to the selective FPR2 antagonist WRW_4_. Following treatment of neutrophils with WRW_4_ (20 μg/ml), migration toward the FPR2 agonist MMK-1 was blocked. In addition, WRW_4_ inhibited the synergistic effect between hrSAA1 (3000 ng/ml) and CXCL8 at 3 and 9 ng/ml by 32% and 53%, respectively (*n* = 3; Figure 9B) thus indicating that hrSAA1 retains its FPR2 binding capacity.

Shape change assays yielded further evidence of this synergistic effect. The net number of activated cells was 3.3-fold higher (*p*-value 0.0079) following stimulation with hrSAA1 (3000 ng/ml) and CXCL8 (3 ng/ml) simultaneously in comparison to the sum of the numbers obtained in response to either stimulus alone (Figure 9C). In addition, measurement of actin polymerization confirmed the findings observed with the Boyden chamber chemotactic assay and the shape change assay. Following stimulation with hrSAA1 and CXCL8 simultaneously, a statistically significant dose-dependent synergistic effect on actin polymerization was observed (relative MFI = 98.8 ± 3.1 for 3000 ng/ml of hrSAA1 and relative MFI = 131.2 ± 7.9 for 1 ng/ml of CXCL8 versus relative MFI = 207.8 ± 23.0 for 3000 ng/ml hrSAA1 + 1 ng/ml CXCL8; *p*-value 0.008; Figure 9D). It was concluded that in three different neutrophil activation assays hrSAA1 retained its synergizing capacity.

## Discussion

In the present study, we purified commercially available rSAA1 expressed in *E. coli*, which has been used in most studies dealing with its biological activities, via RP-HPLC to produce homogenous rSAA1 free of any residual bacterial contaminants. Several biological assays were carried out to determine whether hrSAA1 is a real inflammatory mediator. In contrast to rSAA1, we observed a lack of chemokine induction in response to stimulation of monocytes with hrSAA1 (Table 1). In addition, no ROS production, nor MMP-9 release by monocytes was detected in response to hrSAA1 stimulation. Furthermore, macrophages did not change their expression profile of M1 or M2 markers following incubation with hrSAA1. All these effects have been attributed to rSAA1 binding to TLR2. Nevertheless, hrSAA1 retained its capacity to synergize with CXCL8 in the activation and recruitment of neutrophils, an effect that has also been observed with COOH-terminal fragments of SAA1 (26, 27). This chemotactic activity is reportedly mediated by FPR2 (23–25). By the use of an FPR2 antagonist we previously demonstrated that also synergy in chemotaxis between rSAA1 and CXCL8 is dependent on FPR2 (6). We could confirm here that hrSAA1 still binds to FPR2, as we have observed desensitization of calcium signaling by the FPR2 agonist CCL23(46−137) (51) when FPR2-transfected HEK293 cells were pre-treated with hrSAA1 (data not shown). Moreover, the cooperative interaction between hrSAA1 and CXCL8 in neutrophil chemotaxis is mediated by FPR2. The reported chemotactic activity of SAA1 is rather weak and requires rather high doses, 0.8 μM for monocyte chemotaxis as reported by Su et al. (23) and 4 μM for monocyte and neutrophil chemotaxis as reported by Badolato et al. (10, 52). Furthermore, the synergy between rhSAA1 and CXCL8 we describe in neutrophil actin polymerization already occurs at 300 ng/ml (25 nM) of hrSAA1 (Figure 9D). Therefore, the synergistic interaction of rhSAA1 with other chemoattractants is more relevant than its individual chemotactic property.

**TABLE 1:** Comparison of the biological activities of *E. coli*-expressed SAA1 before (SAA1) and after (hrSAA1) purification to homogeneity using RP-HPLC.

**Activity**	**SAA1**	**hrSAA1**
*In vivo* mononuclear cell attraction	x	x
*In vivo* neutrophil attraction	x	x
Synergy with CXCL8 in neutrophil migration	x	x
Desensitization of FPR2-mediated calcium signaling by CCL23(46−137)	x	x
Induction of MMP-9 release in monocytes	x	–
Induction of chemokine expression in monocytes	x	–
Induction of ROS production in monocytes	x	–
Monocyte survival	x	x
Macrophage polarization	x	–

*x, biological activity demonstrated; – not active.*

The previously reported TLR2-mediated activities of rSAA1, for instance induction or upregulation of cytokine production by rSAA1, are probably due to contaminating bacterial lipoproteins present in the recombinantly produced SAA. Indeed, similar to Burgess et al. (32), we have observed partial downregulation of chemokine induction by monocytes in response to rSAA1 following LPL treatment. Besides lipoproteins, rSAA1 might contain additional chemokine-inducing substances, e.g., non-lipopeptides, such as outer membrane protein A (ompA) or LPS, which were completely removed from rSAA1 by RP-HPLC, but remained unaffected by incubation of rSAA1 with LPL. Indeed, analysis of rSAA1 expressed in *E. coli* revealed the presence of of 91 proteins of bacterial origin. Some of these proteins may also play a role in the inductive capacity of rSAA1 (32). Nevertheless, He et al. have attributed rSAA1-induced CXCL8 production in neutrophils to the activation of FPR2, because the CXCL8 production was inhibited by pertussis toxin (53). On the other hand, we did not observe any CXCL8 production by monocytes treated with up to 1 μM hrSAA1. Our results provide an explanation for the reported discrepancies in the activity of rSAA1 and SAA derived from inflammatory plasma. In contrast to rSAA1, plasma-derived SAA was not found to possess characteristics of an inflammatory mediator (54). These findings are in line with the observations made by Christenson et al. indicating that SAA-rich inflammatory plasma derived from patients with arthritis failed to activate neutrophils (55).

It has long been known that SAA is a lipophilic protein as it can replace apolipoprotein A-I in high density-lipoprotein during an inflammatory insult (56). Taking into consideration the propensity of SAA1 to bind hydrophobic molecules could explain the difficulty encountered to purify rSAA1 from contaminating bacterial products, particularly LPS and lipoproteins. The capacity of SAA to bind hydrophobic molecules is largely intertwined with several of its functions, including its antimicrobial function. Via its interaction with the hydrophobic vitamin retinol, recombinant human SAA1 and recombinant mouse SAA1/3 indirectly regulate the immune response during acute infection (57). Via its binding to ompA of gram-negative bacteria, serum-derived SAA1 acts as an opsonizing agent thereby promoting phagocytosis (15). Through its hydrophobic interaction with LPS, SAA1 dampens the inflammatory response providing protection against excessive inflammatory tissue damage. In LPS-induced acute lung injury, SAA1-transgenic (Tg) mice displayed reduced neutrophil infiltration and lowered expression of inflammatory cytokines (e.g., IL-6 and TNF-α) (58). In a colitis mouse model, SAA1/SAA2 double knockout mice displayed increased weight loss, histological disease scores and TNF-α expression, suggesting that SAA1/SAA2 may provide protection at the intestinal epithelial barrier through its direct antibacterial properties (20).

The idea that SAA is an anti-inflammatory mediator is certainly one worth contemplating. Indeed, a few other studies have provided evidence that SAA mediates anti-inflammatory functions *in vivo*. Murdoch et al. demonstrated that although SAA is essential in zebrafish for the recruitment and maturation of neutrophils, it also plays a role in confining neutrophil-mediated inflammation via the reduction of neutrophil bactericidal activity and expression of inflammatory markers (19). The role of mouse SAA in relation to *in vivo* macrophage polarization was recently investigated in the context of carbon tetrachloride-induced hepatic injury where an increase in fibrogenesis was observed following SAA neutralization. It was found that SAA provided a protective effect via the polarization of macrophages toward an M2b phenotype (59).

Contrary to the observations made by Burgess et al., using rSAA1 derived from an eukaryotic source (32), a number of studies have provided both *in vitro* and *in vivo* evidence regarding the role of SAA in the development of the Th17 response. Through the use of SAA1/2 double knock out mice, Sano et al. demonstrated the role of this acute phase protein in the expression of Th17 cytokines (IL-17A and IL-17F) and the proliferation of Th17 cells in the terminal ileum following segmented filamentous bacteria colonization. These findings were confirmed *in vitro* using recombinant murine SAA1 (rmSAA1). Indeed, the treatment of RORγt^+^ CD4^+^ T cells in suboptimal Th17 polarizing conditions with rmSAA1 induced the expression of both IL-17A and IL-17F. Furthermore, rmSAA1 was found to promote the differentiation of Th17 cells through the upregulation of the RORγt pathway (60). Lee et al. recently made similar observations using rmSAA1 which was found to promote Th17 differentiation of naïve murine CD4^+^ T cells evidenced through the upregulation of several Th17 markers such as IL-23R and S100a4. *In vivo* evidence for the role of murine SAA in the Th17 response was provided using an autoimmune encephalomyelitis mouse model. In this model the Th17 response was diminished in the central nervous system of SAA3 knockout mice in comparison to control wildtype mice (61).

The true nature of SAA, first identified in the late 1970s, remains an enigma (62). Indeed, a whole spectrum of divergent biological activities has been attributed to SAA1 and many apparently contradictory data have been published. Nonetheless, the multiple biological activities attributed to SAA1 could possibly be explained by the fact that SAA1 is an intrinsically disordered protein and thus displays various conformations depending on environmental conditions such as pH or ligand concentration (63). Ji et al. investigated the role of SAA1 in T cell-mediated hepatitis through the use of SAA1-Tg mice; SAA1 was shown to promote hepatic injury through several pathways amongst which the upregulation of chemokine expression (64). This study and the data of Burgess *et al.*, strongly contest the TLR2- and TLR4-mediated *in vitro* activities ascribed to rSAA1. Although TLR activation by rSAA1 is currently rather excluded, it was not implausible. Indeed, several endogenous TLR2/4 ligands exist including proteins and peptides such as β-defensin 2, biglycan, fibronectin, S100 proteins, and high mobility group box 1 (HMGB1) to name a few (65). In contrast, the FPR2-mediated chemotactic activities were confirmed with hrSAA1 and were also observed with synthetic SAA1-derived COOH-terminal peptides which were free of any bacterial components (26, 27), suggesting that this COOH-terminal part of hrSAA1 is responsible for the chemotactic activity of homogenous intact SAA1. Similar to TLRs, FPR2 is activated by a diverse set of endogenous molecules including fatty acids (lipoxin A4), proteins (annexin 1) and peptides (humanin), including chemokine-derived peptides, such as CCL23(46−137) (66, 67).

Some remaining biological activities ascribed to SAA1, such as suppression of antibody production or inhibition of platelet activation and aggregation should be further investigated using homogenous SAA1 (2, 45). Alternatively, the *in vitro* use of rSAA1 produced by eukaryotic expression and the SAA1, SAA2, and SAA3 knock out and/or transgenic animals (19, 20, 58) are important tools to further establish the biological functions of the SAA family members.

## Data Availability Statement

The datasets generated for this study are available upon reasonable request to the corresponding author.

## Ethics Statement

The animal study was reviewed and approved by the animal Ethics Committee of the Federal University of Minas Gerais (295/2018).

## Author Contributions

SA, JV, and SS wrote the manuscript. SA, MDBo, NB, VO, MDBu, MG, and SS performed the experiments and analyzed the data. FA analyzed and supervised the *in vivo* experiments. PP and JV gave technical advice regarding the purification of rSAA1. FA, PP, JV, and SS designed the experiments and provided the funding. All authors corrected the manuscript and approved its final version.

## Conflict of Interest

The authors declare that the research was conducted in the absence of any commercial or financial relationships that could be construed as a potential conflict of interest.
